# Computational Identification and Anti-Inflammatory Evaluation of T19093 as a TLR4/MD2 Inhibitor

**DOI:** 10.2174/0115680266345918250212144023

**Published:** 2025-02-18

**Authors:** Kuida Chen, Ke Shi, Tong Jin, Shipeng Lu, Wu Yin

**Affiliations:** 1 State Key Laboratory of Pharmaceutical Biotechnology, College of Life Sciences, Nanjing University, Nanjing 210023, China;; 2 Medical Research Center, Northern Jiangsu People's Hospital, Yangzhou, Jiangsu 225001, China

**Keywords:** Inhibitor, Machine learning, Virtual screening, TLR4/MD2, Anti-inflammatory, Molecular docking

## Abstract

**Background:**

The TLR4 (Toll-like receptor 4)/MD2 (Myeloid differentiation protein-2) is a crucial target for developing novel anti-inflammatory drugs. Nevertheless, current inhibitors often have significant adverse effects, necessitating the discovery of safer alternatives.

**Objective:**

The investigation aims to identify novel TLR4/MD2 inhibitors with potential anti-inflammatory activity using machine learning and virtual screening technology.

**Methods:**

A machine-learning model was created using the MACCS (Molecular ACCess Systems) key fingerprint. Subsequently, virtual screening and molecular docking were used to evaluate candidate compounds' binding free energy to the TLR4/MD2 complex. Furthermore, ADMET (absorption, distribution, metabolism, excretion, and toxicity) prediction was used to assess the druggable properties of compounds. The most promising compound, T19093, was considered for molecular dynamic simulation. Finally, the anti-inflammatory efficacy of T19093 was further validated using LPS-treated THP-1 cells.

**Results:**

T19093, a polyphenolic compound isolated from the *Gnaphalium* plant genus, showed strong binding to key residues of the TLR4/MD2 complex, with a docking score of -11.29 kcal/mol. Furthermore, ADMET predicted that T19093 has good pharmacokinetic properties and balanced physicochemical properties. Moreover, molecular dynamics simulation confirmed stable binding between T19093 and TLR4/MD2 complex. Finally, it was found that T19093 alleviated LPS-induced inflammatory response by inhibiting the activation of TLR4/MD2 downstream signaling pathways and disrupting the TLR4/MD2 interaction.

**Conclusion:**

T19093 was discovered as a potential novel TLR4/MD2 inhibitor using machine learning and virtual screening techniques and showed potent anti-inflammatory activity, which could provide a new therapeutic alternative for the treatment of inflammation-related diseases.

## 1 INTRODUCTION

TLR4/MD2 is a key receptor complex that recognizes and binds to pathogen-associated molecular patterns (PAMPs) and triggers host immune responses, playing an important role in inflammatory signaling [1, 2]. The activated TLR4/MD2 complex produces inflammatory mediators such as TNF-α, IL-6, and IL-1β through MyD88-dependent and TRIF-dependent signaling pathways, impacting host defense and immune response [3-5]. Studies have shown that prolonged or excessive activation of TLR4/MD2 can result in various inflammation-related conditions, such as sepsis, atherosclerosis, and autoimmune diseases [6]. In sepsis, TLR4/MD2 activation not only leads to systemic inflammatory response syndrome (SIRS) but also triggers immune paralysis, increasing the patient's susceptibility to secondary infections [7]. Therefore, TLR4/MD2 is a promising target for the enhancement of new anti-inflammatory drugs.

Presently, several TLR4/MD2 inhibitors have been formulated, including Tak-242, Eritoran, and FP7 [8-10]. However, clinical trials often show serious side effects from these inhibitors. Tak-242 may cause hepatotoxicity and immunosuppression [11], while Eritoran's efficacy is unproven. Thus, innovative and more powerful TLR4/MD2 inhibitors are needed to overcome present drug limitations. Natural products have emerged as a valuable source of drug development due to their structural variety and low toxicity. Curcumin and resveratrol have been shown to reduce LPS-induced inflammation by lowering TLR4 expression and inhibiting its signal transduction [12, 13]. Additionally, quercetin inhibits TLR4 signaling by affecting the formation of the TLR4/MD2 complex [14].

Due to significant advances in computer technology and chemical simulation theory, computational techniques are often utilized in drug development to speed up experimental screening. Virtual screening can choose a few likely active compounds from many known compounds, reducing experimental verification [15]. Machine learning techniques quickly discover active chemicals, while molecular docking and molecular dynamics simulations demonstrate their binding and stability [16]. The multi-stage virtual screening method has identified many biologically active chemicals. Combinatorial peptide libraries were screened using molecular dynamics simulation and binary QSAR (Quantitative Structure-Activity Relationship) model to identify α-glucosidase inhibitors [17]. A tetrapeptide library was used to uncover new µ-opioid receptor inverse agonists using structure-based virtual screening [18]. Additionally, machine learning technology is common in developing novel antibacterial and antiviral medications [19, 20].

Our research aims to screen for novel TLR4/MD2 inhibitors with potential anti-inflammatory activity. Using machine learning and virtual screening technology to significantly improve screening efficiency and accuracy, we successfully discovered the potential TLR4/MD2 inhibitor T19093 and verified its anti-inflammatory activity. Moreover, this study not only took advantage of the structural diversity and low toxicity of natural products but also ensured that the selected compounds had high binding energy and stability, filling the gap in the safety and efficacy of existing TLR4/MD2 inhibitors.

## 2 MATERIALS AND METHODS

### 2.1 Data Curating Process

TLR4/MD2 targeting compounds were acquired from ChEMBL (Chemical Database of Bioactive Molecules) [21], duplicates were eliminated, and the data was kept focused on human TLR4/MD2. The compounds were classified as either active (0) or inactive (1). The dataset consisted of 662 active and 701 inactive compounds, with 80% allocated for training and 20% for testing. Principal component analysis (PCA) [22, 23] was used to explore the chemical space. PCA compressed the feature space from high-dimensional data to 3 dimensions, retaining most of the variance and removing redundant information. Additionally, PCA was used for data visualization, mapping the compounds onto 2D and 3D spaces to clearly illustrate the distribution of samples and distinguish between positive and negative samples based on chemical diversity.

### 2.2 Feature Engineering for Molecular Representation

Molecular fingerprinting explains features by converting molecular structures into binary codes. MACCS, PubChem, and Morgan fingerprints are examples of common types [24, 25]. We employed 166 structural feature-based MACCS fingerprints for molecular representation in machine learning. Learning curves and the recursive feature elimination (RFE) algorithm were utilized to address overfitting and dimensionality concerns, ensuring optimal feature selection.

### 2.3 Building and Evaluating Machine Learning Models

Machine learning models were developed using four common classification algorithms: Decision Tree (DT) [26], Random Forest (RF) [27], Support Vector Machine (SVM) [28], and Naive Bayes (NB) [29]. The models were implemented using MACCS fingerprint descriptors in Python’s Scikit-learn library [30, 31], with hyperparameter optimization performed using GridSearchCV to find the optimal parameter combinations [32]. Model performance was evaluated based on accuracy, precision, recall, F1 score, and area under the curve (AUC) [33]. For Decision Tree (DT), the maximum depth (max_depth) was optimized with values of 10 and 20 to prevent overfitting. In Random Forest (RF), two key hyperparameters, the number of trees (n_estimators) and max_depth were tested with values of 100 and 200 for trees, and 10 and 20 for depth. Support Vector Machine (SVM) was optimized by tuning the regularization parameter (C) and kernel coefficient (gamma) with values of 0.1 and 1, and 0.01 and 0.1, respectively. Naive Bayes (NB), being a simple probabilistic model, was not hyperparameter-tuned. For all models except NB, GridSearchCV was used to optimize parameters, with a parameter grid defined for each model:

param_grid = {

'RandomForest': {'classifier__n_estimators': [100, 200], 'classifier__max_depth': [10, 20]},

'SVM': {'classifier__C': [0.1, 1], 'classifier__gamma': [0.01, 0.1]},

'DecisionTree': {'classifier__max_depth': [10, 20]},

'NaiveBayes': {}

}

Models were evaluated on a 20% hold-out test set, and performance was assessed using accuracy, precision, recall, F1 score, and ROC AUC.

### 2.4 Molecular Docking

The LigPre module of Maestro 11.9 [34] was employed to protonate and minimize the compound library, which was drawn from the TargetMol natural product library (19377 compounds). The OPLS3e force field [35] was utilized to achieve this and process the TLR4/MD2 target protein structure (PDB ID: 4G8A) on the Maestro 11.9 platform, which included the removal of water and ions, protonation, filling in missing sections, and energy minimization. The Glide module of Schrödinger Maestro [36] was employed to conduct virtual screening, and the Protein Preparation Wizard module was employed to perform protein pretreatment. The docking site was based on the natural ligand LPS of the protein and was set to the center of mass of a 15 Å box (x = -20.76, y = -17.40, z = -16.87).

### 2.5 Molecular Dynamics

AMBER 22 [37] was used to run all-atom simulations of the small molecule-protein combination. The HF SCF/6-31G* in Gaussian 09 [38] was used to compute small molecule charges using the force fields ff14SB and GAFF2 [39, 40]. The system was heated from 0 K to 298.15 K over 200 ps after energy optimization. Next came 500 ps NVT and 500 ps NPT equilibration, and lastly a 100 ns NPT simulation. Non-bonded interactions were cut off at 10 Å, long-range electrostatics were computed with PME [41], and hydrogen bonds were constrained using SHAKE, and the temperature was controlled with the Langevin algorithm (γ=2 ps^-1) [42]. Trajectories were saved every 10 ps. The protein-ligand binding free energy for all systems was calculated using the MM/GBSA methods [43]. The MD trajectory from 45-50 ns was utilized for calculations, using the following formula:


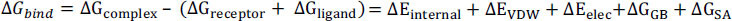


### 2.6 Cell Culture Sources and Conditions

THP-1 cells were purchased from ATCC (American Type Culture Collection) and cultured in RPMI 1640 medium with 10% FBS and 1% penicillin/streptomycin, incubated at 37°C with 5% CO_2_.

### 2.7 Quantitative Real-time PCR

Total RNA was extracted from cells using Trizol reagent (Invitrogen, CA, USA) according to the manufacturer's instructions. cDNA was synthesized from 1 μg of RNA using the High-Capacity cDNA Reverse Transcription Kit (Applied Biosystems, CA, USA). The mRNA expression levels of specific target genes were analyzed by real-time PCR with SYBR Green Master Mix (Bio-Rad, CA, USA) on a Bio-Rad C1000 Real-Time PCR Detection System. The target genes analyzed included TNF-α, IL-6, IL-1β, and CXCL2, and the expression levels were quantified using the 2−ΔΔCt method, with β-actin serving as the housekeeping gene for normalization. Primer sequences for RT-qPCR are provided in Supplementary Table **1**. All reactions were performed in triplicate, and data were analyzed using the Bio-Rad CFX Manager software.

### 2.8 Western Blot and Co-immunoprecipitation (Co-IP)

For protein extraction, cells were lysed in RIPA buffer (Thermo Fisher Scientific, MA, USA) with protease inhibitors (Roche, Germany) for 30 minutes on ice. Protein concentration was determined using the BCA protein assay kit (Thermo Fisher Scientific). For Western blotting, equal amounts of protein (20–40 μg) were separated by SDS-PAGE and transferred to PVDF membranes (Millipore, MA, USA). After blocking with 5% BSA in TBS-T for 1 hour, membranes were incubated overnight with primary antibodies targeting p-p65, p65, p-ERK, ERK, p-JNK, JNK, p-P38, P38, TLR4, MD2 and GAPDH. Following incubation with HRP-conjugated secondary antibodies, protein bands were detected using LumiGLO chemiluminescence reagent (Cell Signaling Technology, MA, USA) and imaged with the ChemiDoc XRS+ Imaging System (Bio-Rad). For co-immunoprecipitation, lysates were incubated with specific antibodies overnight at 4°C, followed by incubation with protein A/G agarose beads (Santa Cruz Biotechnology, CA, USA). After washing, immune complexes were eluted and analyzed by SDS-PAGE and Western blotting. The primary antibodies used for WB and Co-IP included anti-TLR4 and anti-MD2.

### 2.9 Cell Viability Assay

THP-1 cells in 96-well plates were treated with 20 µl/well MTT solution (4 mg/ml). After 4 hours of incubation at 37°C, the supernatant was removed. DMSO was then added at 200 µl/well for 10 minutes. Absorbance was measured at 570 nm using a BioTek FL × 800 fluorescence microplate reader (Winooski, VT, USA).

### 2.10 Immunofluorescence

THP-1 cells were fixed with 4% paraformaldehyde, permeabilized with 0.25% Triton X-100 for 30 minutes, and blocked with 1% BSA. The cells were then incubated overnight at 4°C with a P65 antibody, followed by washing and incubation with a FITC-conjugated secondary antibody. Nuclei were counterstained with DAPI, and the samples were imaged using a Leica confocal microscope (Leica, Wetzlar, Germany; Solms).

### 2.11 Statistical Analysis

Data analysis was performed using GraphPad Prism and results are presented as mean ± SEM. One-way ANOVA with Tukey's post hoc test was used for multiple group comparisons and the student’s t-test for two groups. Statistical significance was set at P<0.05, indicated as *P<0.05, **P<0.01, and ***P<0.001.

## 3 RESULTS

### 3.1 Chemical Diversity Analysis

**Fig. (1):**
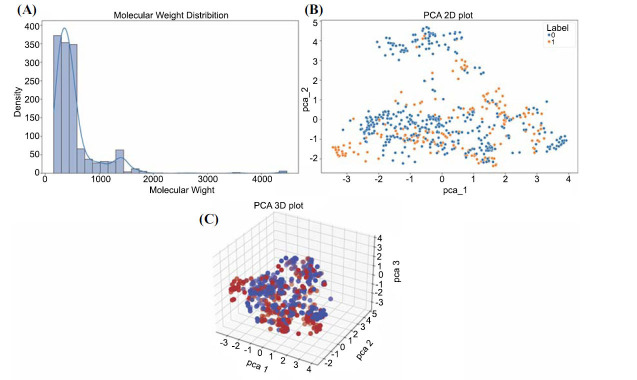
Molecular diversity analysis and PCA of active and inactive compounds. (**A**) Histogram of the molecular weight distribution of compounds in the dataset. (**B**) The two-dimensional PCA map depicts the distribution of 662 active compounds (blue dots) and 701 inactive compounds (orange dots) following dimensionality reduction. (**C**) The three-dimensional PCA plot displays inactive chemicals (red points) and active compounds (blue points).

The composition of the modeling dataset significantly impacts the accuracy of the machine-learning algorithm. MACCS fingerprints were chosen as molecular descriptors for the compounds in the training set. The histogram shows the molecular weight distribution of the chemicals in the dataset. Most compounds range from 200 to 600 Da, with some between 800 and 1500 Da, and a few over 2000 Da, indicating a diverse and broad distribution of molecular weights (Fig. **1A**). PCA reduced the features of 662 active and 701 inactive compounds to two dimensions. While the two types are somewhat separated, there is overlap, highlighting the diversity of chemical structures in the dataset (Fig. **1B**). The 3D PCA plot shows the distribution of active and inactive chemicals in three-dimensional space, with a clear separation between them. This highlights the chemical diversity in the dataset (Fig. **1C**) and suggests that the two classes have distinguishable features.

### 3.2 Development and Validation of Machine Learning Models

**Fig. (2):**
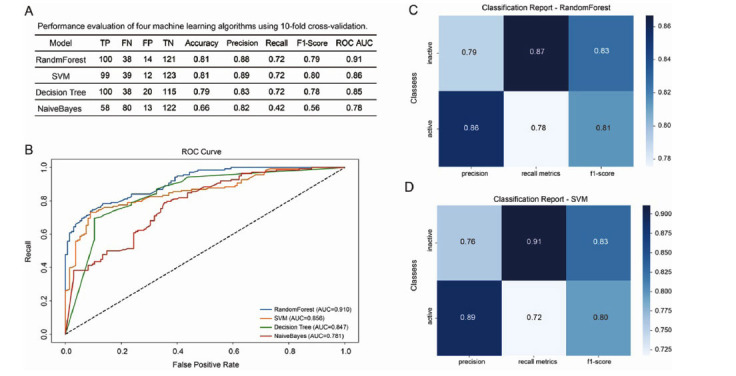
Performance evaluation and classification reporting of machine learning algorithms. (**A**) RF, SVM, DT, and NB in 10-fold cross-validation, encompassing the counts of true positives (TP), false negatives (FN), false positives (FP), and true negatives (TN), along with metrics such as accuracy, precision, recall, F1-score, and the area under the ROC curve. (**B**) The ROC curve shows the performance of four machine learning models. (**C** & **D**) Classification report for the RF and SVM model showing precision, recall, and F1 metric in classifying active and inactive compounds.

Four prediction models (RF, SVM, DT, and NB) were developed and evaluated using 10-fold cross-validation and independent validation. In the test set of 273 compounds, the correct classifications (TP + TN) were 221 for RF, 222 for SVM, 215 for DT, and 180 for NB. RF and SVM showed the highest accuracy, precision, recall, and F1 scores (Fig. **2A**). In addition, receiver operating characteristic (ROC) curves and calculated AUC were used to assess model performance. RF and SVM with AUC values of 0.910 and 0.856, respectively, followed by DT at 0.847 and NB at 0.781 (Fig. **2B**). This indicates that RF and SVM are the most effective at classifying compounds in the dataset. To further validate model performance, we tested an independent dataset under the same conditions. RF and SVM models showed higher recall and F1 scores for the inactive class and greater precision for the active class (Fig. **2C** and **2D**). These results confirm that RF and SVM performed well on both the training and test datasets, demonstrating strong generalization and robustness.

### 3.3 Virtual Screening and Docking Analysis of TLR4/MD2 Inhibitors

**Fig. (3):**
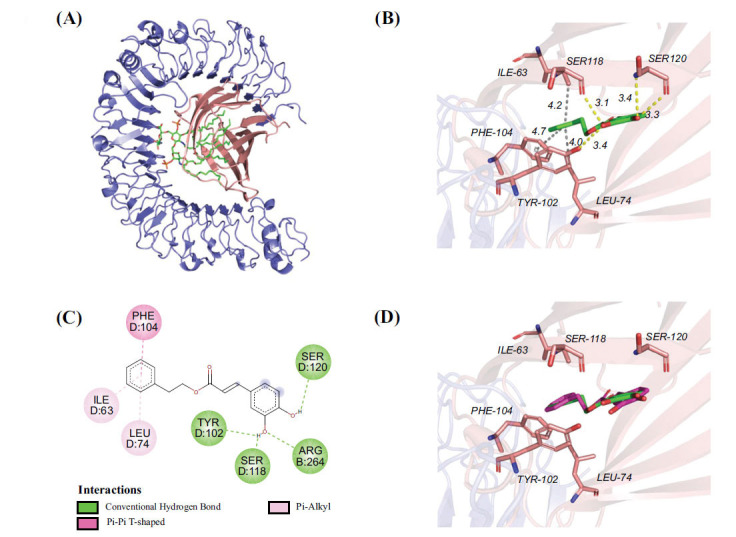
The docking of CAPE with TLR4/MD2. (**A**) The complete 3D structure of the TLR4/MD2 (PDB ID: 4G8A). (**B**) A detailed view of the active site where the ligand is bound. (**C**) The 2D diagram showed the interactions between the protein and the ligand in the TLR4/MD2 complex. (**D**) The re-docking outcomes of LPS (highlighted in red) with the TLR4/MD2 complex.

The RF model with excellent overall performance was used to preliminarily screen 19,377 compounds in the natural product database, and 1897 TLR4/MD2 candidate inhibitors with a model score greater than 0.8 were obtained. The active site of TLR4/MD2 consists mainly of ILE-63, SER-118, PHE-104, TYR-102, LEU-74, SER-120, ARG-264 and ILE-117. The binding mode of CAPE, a positive inhibitor of the TLR4/MD2 shown in (Fig. **3A**). CAPE may engage in robust hydrogen bonding interactions with the key residues SER-120, SER-118, and TYR-102. In addition, the benzene ring of the CAPE forms a pi-pi conjugated interaction with PHE-104 (Fig. **3B** and **3C**). To evaluate the effectiveness of the binding mode and method, the known original ligand was docked into the binding site of TLR4/MD2. The binding pose showed strong overlap with the ligand in the previous complex (Fig. **3D**), suggesting that the screening method was effective and rational.

Docking studies were conducted on compounds identified by the machine learning model using Schrödinger software, and two rounds of screening were performed on potential compounds with SP (Standard Precision) and XP (Extra Precision) less than -6. These compounds were ranked based on their MMGBSA (Molecular Mechanics Generalized Poisson-Boltzmann Surface Area) calculated binding energy less than -40.0 kcal/mol, binding mode, and visual inspection. Table **1** displays the outcomes for the top 10 compounds based on their binding energy.

**Fig. (4):**
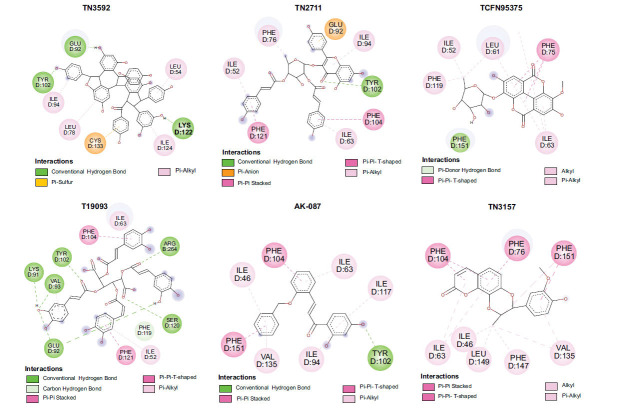
Molecular docking of selected compounds with TLR4/MD2 complex. Candidate compounds (TN3592, TN2711, TCFN95375, T19093, AK-087, TN3157, TN5516, STOCK1N-28227, T13966, and T5156) target TLR4/MD2 active sites, the main forces include conventional hydrogen bonds (green), Pi-sulfur bonds (yellow), Pi-stacking (pink) and Pi-alkyl (purple).

The docking diagrams of the top 10 compounds with key residues in the TLR4/MD2 were displayed (Fig. **4** and Fig. **S1**). The individual candidate compounds showed different binding potentials. For instance, TN3592 enhanced its binding stability to the target by forming hydrogen bonds with GLU-92, TYR-102, and LYS-122, in addition to forming Pi-sulfur interactions with CYS-133. Similarly, TN2711 stabilized its binding in the binding site by forming hydrogen bonds with TYR-102 and forming Pi-Pi stacking interactions with PHE-104 and PHE-121. On the other hand, T19093 was particularly prominent, as it showed the strongest binding stability and potential by forming multiple hydrogen bonds with various key residues, including TYR-102, GLU-92, VAL-93, ARG-264, LYS-91, and SER-120, while also forming Pi-Pi stacking interactions with PHE-104 and PHE-121, making it a very promising TLR4/MD2 inhibitor. In contrast, AK-087 exhibited good binding ability by forming hydrogen bonds with TYR-102 as well as Pi-Pi stacking interactions with PHE-104 and PHE-151. Furthermore, TN5516 enhanced its binding stability through hydrogen bonding interactions with TYR-102 and ASN-339 along with Pi-Pi stacking interactions with PHE-104. Finally, T5156 showed promise as a potential inhibitor by forming hydrogen bonds with TYR-102 and Pi-Pi stacking interactions with PHE-76. These compounds demonstrated their ability to act as potential inhibitors in the TLR4/MD2 complex through different hydrogen bonding and Pi-Pi interactions.

### 3.4 ADMET Analysis

To determine the suitability of compounds as drugs, the SwissADME online server (http://www.swissadme.ch) and OSIRIS Property Explorer software were used to evaluate the pharmaceutical properties and safety characteristics of 10 compounds. Figs. (**5** and **S2**) present the drug scores of the 10 candidate compounds along with their corresponding radar charts. T19093 performed well in multiple attribute dimensions (such as polarity, flexibility, and solubility), demonstrating its potential stability and adaptability in biological environments. In contrast, although TCFN95375 scored the highest, it lacked flexibility and solubility, and T13966 was slightly insufficient in polarity and lipid solubility, which may affect its overall binding ability and stability in the binding pocket. Notably, T19093, a polyphenolic compound known as 2,3,4,5-tetracaffeoyl-D-glucaric acid, is a caffeoyl-D-glucuronic acid derivative isolated from the plant genus *Gnaphalium* and is recognized for its potential pharmacological benefits [44]. Furthermore, T19093 exhibited diverse interaction types, good physicochemical properties, and excellent performance in biological activity and binding stability, making it the most promising candidate. Consequently, T19093 was finally selected as the preferred TLR4/MD2 inhibitor.

### 3.5 Stability of T19093 Binding to TLR4/MD2

**Fig. (6):**
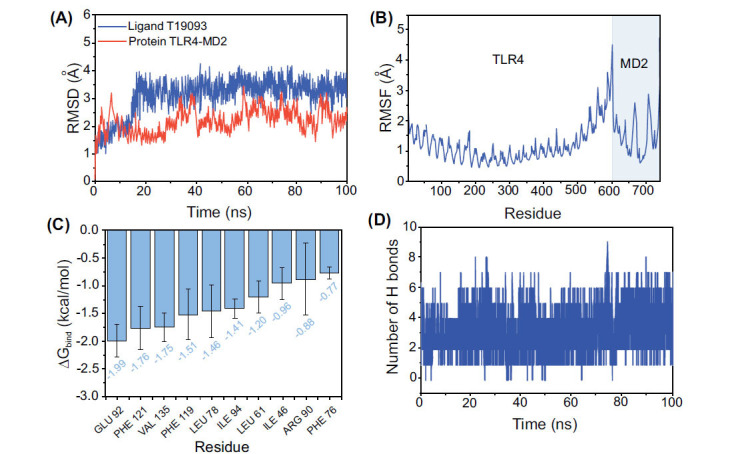
TLR4/MD2_T19093 molecular dynamics simulation. (**A**) RMSD of the complex over time during the molecular dynamics simulation. (**B**) RMSF is calculated based on the molecular dynamics simulation trajectory. (**C**) The 10 key amino acids involved in the TLR4/MD2_T19093 interaction. (**D**) The count of hydrogen bonds between TLR4/MD2_T19093 during the simulation.

A 100 ns molecular dynamics simulation was conducted to evaluate the stability of T19093 binding to the TLR4/MD2 complex. RMSD (Root Mean Square Deviation) trajectory showed that the TLR4/MD2_T19093 reached convergence after 20 ns of simulation. It then fluctuated in the range of 2.5-4 angstroms (Fig. **6A**). The RMSF (Mean Square Fluctuation) of the protein was within 2 Å, indicating a rigid main structure, possibly due to interaction with T19093 (Fig. **6B**). Using the MM/GBSA method, the binding energy of TLR4/MD2_T19093 was calculated to be -27.74±2.07 kcal/mol based on the molecular dynamics’ simulation trajectory (Table **2**). Energy decomposition revealed that van der Waals forces primarily drive the binding of TLR4/MD2_T19093 and identified the top 10 amino acids involved in binding T19093 to the TLR4/MD2 complex (Fig. **6C**). In addition, the number of hydrogen bonds during the simulation ranged from 0 to 9, with 3 to 4 being the majority, indicating that hydrogen bonding is crucial for the stable binding of TLR4/MD2_T19093 (Fig. **6D**). These results indicate that T19093 has a high stability in binding to the TLR4/MD2 complex.

### 3.6 T19093 Reduces Inflammatory Responses and Blocks TLR4/MD2 Signaling

**Fig. (7):**
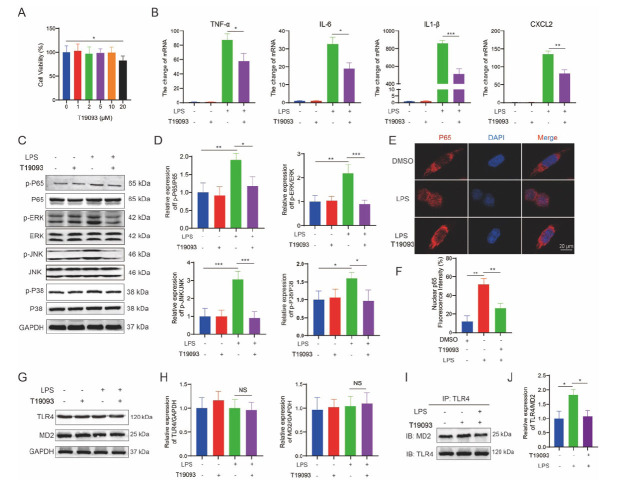
T19093 prevents the production of inflammatory factors and disrupts TLR4/MD2 signaling. (**A**) THP-1 cell viability after exposure to varying concentrations of T19093. (**B**) mRNA levels of TNF-α, IL-6, IL-1β, and CXCL2 were assessed in THP-1 cells treated with LPS, T19093, or their combination. (**C** & **D**) Protein levels of p-P65, p-ERK, p-JNK, and p-P38 were analyzed in THP-1 cells treated with LPS, T19093, or their combination. (**E** & **F**) Immunofluorescence staining was performed to observe the localization of P65 and DAPI in THP-1 cells. The quantitative results of fluorescence intensity are shown. (**G** & **H**) Immunoblotting was conducted to assess the TLR4 and MD2 levels in THP-1 cells. The quantitative results of Western blot were shown. (**I** & **J**) Western blot analyses of TLR4 co-immunoprecipitating with MD2 were measured in the THP-1 cells with LPS and T19093 alone or combination. The quantitative results of Western blot were shown. Data are expressed as means ± SEM. *P<0.05, **P<0.01, ***P<0.001.

To verify the anti-inflammatory ability of T19093, a cellular inflammatory injury model was established using LPS-treated THP-1 cells. First, the effect of different doses of T19093 on viability was determined. T19093 significantly reduced cell survival and exhibited clear cytotoxic effects at a concentration of 20 μM (Fig. **7A**). To minimize cytotoxicity, a lower dose of 10 μM T19093 was used to treat the cells. T19093 significantly reduced the LPS-induced upregulation of pro-inflammatory mRNA levels, including TNF-α, IL-6, IL-1β, and CXCL2 (Fig. **7B**). Western blot revealed that LPS stimulation significantly increased the phosphorylation levels of p65, ERK, JNK, and P38 in THP-1 cells, but this effect was reversed by T19093 treatment (Fig. **7C** and **7D**). In addition, immunofluorescence showed that p65 translocated to the nucleus in response to LPS stimulation, however, nuclear translocation of p65 was significantly inhibited by treatment with T19093 (Fig. **7E** and **7F**). T19093 did not significantly affect TLR4 and MD2 protein levels following LPS stimulation (Fig. **7G** and **7H**). Interestingly, the interaction between TLR4 and MD2 increased in response to LPS stimulation but was significantly diminished with T19093 treatment (Fig. **7I** and **7J**). These findings suggest that T19093 may play a role in regulating inflammatory responses.

## 4 DISCUSSION

TLR4/MD2 is a potential anti-inflammatory drug target suitable for the treatment of various inflammatory diseases [6, 45-47]. However, existing TLR4/MD2 inhibitors often have serious adverse reactions, while natural products have become valuable drug resources due to their structural diversity and low toxicity [48, 49]. Due to advancements in computer technology and chemical simulation theory, virtual screening has been widely utilized in drug discovery [50]. In this study, a potential TLR4/MD2 inhibitor T19093 was discovered through machine learning and virtual screening methods, and its anti-inflammatory effect was verified. Notably, T19093 belongs to the class of polyphenolic compounds that have attracted attention for their broad biological activities, particularly in anti-inflammatory and antioxidant aspects, further supporting its efficacy in inhibiting the TLR4/MD2 target.

TLR4/MD2 plays a central role in immune responses to pathogens, making it a critical target for the development of therapies aimed at treating inflammatory diseases [51-54]. Kim *et al*. [55] reported that TLR4 and MD-2 form a heterodimer that recognizes LPS, and Eritoran, an LPS antagonist, inhibits its activity by binding to the TLR4-MD-2 complex. Similarly, it has been reported that the novel β,α-trehalose-type Lipid A mimetics can selectively block the LPS binding site on MD-2, serving as potent TLR4 antagonists and potential antisepsis candidates [56]. Furthermore, novel carboxylate-based glycolipids have been designed to antagonize TLR4 signaling by binding to MD-2, showing promising inhibition of LPS-induced inflammation [57]. The critical role of LYS-91 and GLU-92 in LPS-induced TLR4 and MD2 interaction was identified by Huber *et al* [58]. Moreover, Curcumin Analogue L48H37 can bind to the hydrophobic region of MD2 and form hydrogen bonds with Tyr102 and Arg90 [59]. HDCA (Hyodeoxycholic acid) inhibits TLR4-mediated inflammatory responses by forming a hydrogen bond with Ser120 to prevent LPS from binding to TLR4/MD2 [60]. In our study, we found that T19093 formed multiple interactions with the key residues of TLR4/MD2, GLU-92, VAL-93, TYR-102, PHE-104, and SER-120, thereby stably binding and inhibiting its signal transduction. In this study, THP-1 cells, a human monocytic cell line, were selected to evaluate the anti-inflammatory effects of T19093. THP-1 cells are widely used in immunological research due to their ability to differentiate into macrophage-like cells in response to stimuli such as phorbol 12-myristate 13-acetate [61, 62]. Upon differentiation, these cells exhibit key features of macrophages, including the expression of various pattern recognition receptors (PRRs) such as TLR4 [63, 64], making them an ideal model for studying the modulation of TLR4-mediated inflammation. Given that TLR4/MD2 is crucial in the activation of immune responses, particularly in macrophages and monocytes, the THP-1 cell line serves as a reliable system to investigate the impact of T19093 on the TLR4/MD2 signaling pathway and its downstream inflammatory effects. Furthermore, THP-1 cells are commonly used in screening assays for anti-inflammatory compounds due to their responsiveness to LPS stimulation and their ability to replicate the pro-inflammatory cytokine profile seen in human inflammatory conditions.

In neuroinflammatory disorders, such as neurodegenerative diseases and neuropathic pain, TLR4/MD2 activation in microglia and astrocytes contributes to neuronal damage [65-68]. ACT001 has been identified as a TLR4 antagonist that targets the co-receptor MD2, effectively inhibiting TLR4 signaling and mitigating neuroinflammation [69]. In addition, ITH12674, a melatonin-sulforaphane hybrid, attenuates neuroinflammation by blocking LPS binding to MD2 and inhibiting TLR4 signaling, offering promising therapeutic potential [70]. Therefore, we speculate that T19093 could provide neuroprotective effects by disrupting the TLR4/MD2 interaction, thereby inhibiting downstream inflammatory signaling pathways responsible for neuronal damage. Baicalein has been identified as an MD2 inhibitor, blocking TLR4-MD2 activation and protecting against acute lung injury by suppressing inflammatory signaling [71]. The novel compound Z20, a potential TLR4-MD2 inhibitor, effectively alleviated sepsis-induced organ injury and inflammation by targeting the TLR4-MD2 complex [72]. The therapeutic potential of T19093, as a selective TLR4/MD2 inhibitor, is evident in its ability to modulate immune responses without broad immune suppression, which could reduce the side effects commonly associated with traditional anti-inflammatory drugs. In sepsis and acute lung injury, where TLR4-MD2 signaling exacerbates systemic inflammation, T19093 could serve as a promising therapeutic agent to alleviate organ injury and improve survival rates. Osteoarthritis (OA), where TLR4/MD2 signaling in chondrocytes and synoviocytes plays a role in cartilage degradation and inflammation. 6-shogaol has been shown to prevent cartilage damage and synovial inflammation in osteoarthritis by hindering the interaction between TLR4/MD-2 complex in chondrocytes [73]. Therefore, targeting the TLR4/MD2 complex, T19093 offers a potential disease-modifying treatment for OA. While the current study focused on its anti-inflammatory effects, it is worth noting that compounds targeting TLR4/MD2 signaling have shown promise in modulating immune responses in cancer. TLR4 signaling has been implicated in the tumor microenvironment, where it can influence tumor progression, metastasis, and immune evasion [74-77]. Selective targeting of the TLR4 co-receptor, MD2, using the inhibitor L6H21, has been shown to suppress colon cancer growth and metastasis by blocking the TLR4-MD2/NF-κB signaling pathway [76]. Similarly, Gastrodin attenuates colitis and prevents tumorigenesis in mice by inhibiting TLR4/MD2/NF-κB signaling, offering potential as a therapeutic agent in colitis-associated cancer [74]. Although we did not directly investigate the anticancer properties of T19093 in this study, its inhibitory effects on TLR4/MD2 may suggest potential immunomodulatory benefits in cancer therapy.

While this study provides promising results regarding the potential of T19093 as a TLR4/MD2 inhibitor, several important limitations should be addressed in future research. The findings are primarily based on in silico and *in vitro* approaches. The lack of *in vivo* validation limits our ability to draw definitive conclusions about its effectiveness and safety in living organisms. Future studies should include animal models to assess the pharmacokinetics, pharmacodynamics, and therapeutic efficacy of T19093 in relevant disease models, such as sepsis, neuroinflammation, or autoimmune diseases. Numerous papers have discussed the application of conventional virtual screening methods to the investigation of TLR4/MD2 inhibitors [48, 78]. In this study, we employed a combination of machine learning techniques to greatly enhance the efficiency and accuracy of screening. Compared with commercial and public chemical libraries, we chose natural products as screening objects and used their structural diversity and low toxicity to discover more potential drug candidate molecules. Although the compound dataset used in this study covers a wider range of molecular weight distribution and chemical diversity, the total sample size is relatively limited. Subsequent studies can further improve the training effect and prediction ability of the machine learning model by increasing the sample size, especially compounds from different sources and types.

## CONCLUSION

In summary, T19093, a potential TLR4/MD2 inhibitor, was discovered using machine learning and virtual screening techniques. By binding to key residues of the TLR4/MD2 complex, T19093 disrupts their interaction and inhibits the activation of downstream signaling pathways, thereby demonstrating potent anti-inflammatory activity. T19093 could potentially offer improved efficacy and reduced toxicity compared to existing treatments. This compound shows great promise as a new therapeutic for the treatment of inflammation-related diseases.

## Figures and Tables

**Fig. (5):**
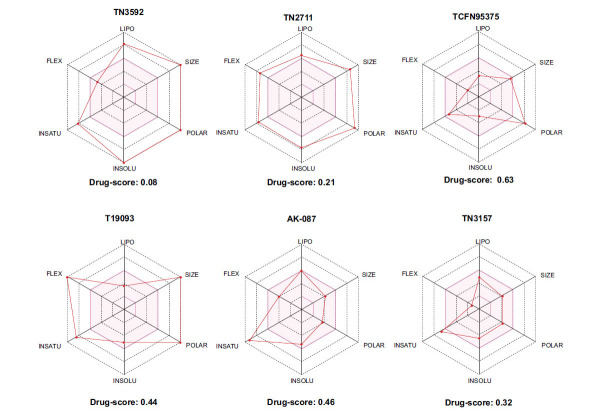
Drug scores of top1-6 candidate compounds and radar chart of their physicochemical properties. SIZE (size), POLAR (polarity), INSATU (unsaturation), INSOLU (insolubility), FLEX (flexibility), and LIPO (lipid solubility). The drug score of each compound is given below the radar chart.

**Table 1:** Virtual screening of natural compound library for TLR4/MD2 target.

**Name**	**MMGBSA**	**Score**	**Structure**	**MW**	**Probability**
TN3592	-82.88	-7.26	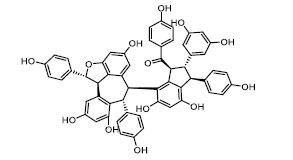	922.94	0.82
TN2711	-68.21	-10.96	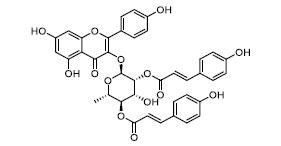	724.67	0.83
TCFN95375	-67.50	-10.98	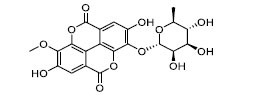	462.36	0.81
T19093	-66.63	-11.29	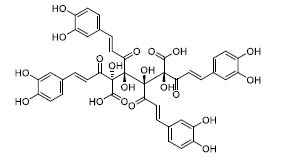	858.71	0.85
AK-087	-65.82	-11.30	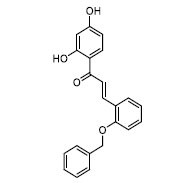	346.38	0.83
TN3157	-65.70	-9.84	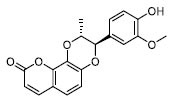	340.33	0.85
TN5516	-65.64	-10.95	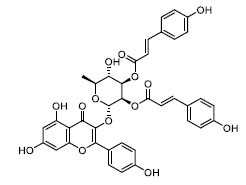	724.67	0.83
STOCK1N-28227	-65.50	-9.36	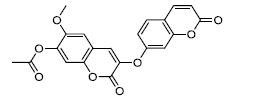	394.33	0.86
T13966	-65.48	-11.82	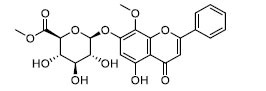	474.42	0.80
T5156	-65.38	-11.03	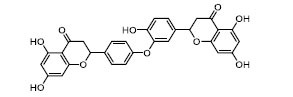	542.50	0.80

**Table 2:** Binding free energies and energy components predicted by MM/GBSA (kcal/mol).

**System Name**	**TLR4-MD2_T19093**
Δ*E*_vdw_	-42.77± 1.79
Δ*E*_elec_	-58.70±12.36
ΔG_GB_	81.25±10.04
ΔG_SA_	-7.51± 0.12
ΔG_bind_	-27.74± 2.07

**Note: ** ΔE_vdW_: van der Waals energy. ΔE_elec_: electrostatic energy. ΔG_GB_: electrostatic contribution to solvation. ΔG_SA_: non-polar contribution to solvation. ΔG_bind_: binding free energy.

## Data Availability

The data supporting the findings of the article are available within the article.

## LIST OF ABBREVIATIONS

AUC: Area under the curve
DT: Decision Tree
PCA: Principal component analysis
SIRS: Systemic inflammatory response syndrome
