# Supplementary material for: Computational Identification and Anti-Inflammatory Evaluation of T19093 as a TLR4/MD2 Inhibitor
Source: Curr Top Med Chem. 2025 Feb 18;25(26):3068–81. doi: 10.2174/0115680266345918250212144023 (PMC12728533; doi:10.2174/0115680266345918250212144023)
Supplement: Supplementary file 1 [file CTMC-25-26-3068_SD1.pdf]

# Supplementary Material

# Computational Identification and Anti-Inflammatory Evaluation of T19093 as a TLR4/MD2 Inhibitor

Kuida Chen[1,#], Ke Shi[2,#], Tong Jin[1], Shipeng Lu[1] and Wu Yin[1,*]

*[1]State Key Laboratory of Pharmaceutical Biotechnology, College of Life Sciences, Nanjing University, Nanjing 210023, China; [2]Medical Research Center, Northern Jiangsu People's Hospital, Yangzhou, Jiangsu 225001, China*

## SUPPLEMENTARY MATERIALS AND METHODS

### Drugs, Chemicals, and Antibodies

2,3,4,5-tetracaffeoyl-D-glucaric acid (Cat # B28262) was acquired from Yuanye Biotech Co., Ltd. (Shanghai, China), while Lipopolysaccharide (LPS, Cat # L2880) was obtained from Sigma-Aldrich Co., Ltd. (St. Louis, MO, USA).Various antibodies were obtained from Abmart Co., Ltd. (Shanghai, China), including NF-κB p65 Antibody (1:1000, Cat # T55034), Phospho-NF-κB p65 (Ser536) Antibody (1:1000, Cat # TP56372), p38 Antibody (1:1000, Cat # T55600), Phospho-p38 (Thr180/Tyr182) pAb (1:1000, Cat # T40076), ERK1/2 Antibody (1:1000, Cat # T40071), Phospho-ERK1/2 (Tyr204) Antibody (1:1000, Cat # TP56192), JNK1/2/3 Antibody (1:1000, Cat # T40073), and Phospho-JNK1/2/3 (Thr183+Tyr185) Antibody (1:1000, Cat # TA3318). Secondary antibodies, such as Goat Anti-Mouse IgG-HRP (1:5000, Cat # M21001) and Goat Anti-Rabbit IgG-HRP (1:5000, Cat # M21002), were also sourced from Abmart Co., Ltd.

**Supplementary Table 1. Primer sequences used in RT-qPCR.**

| Name | Sequence | Purpose |
|---|---|---|
| Human-IL-1β-for | TTCGACACATGGGATAACGAGG | Quantitative PCR for human IL-1β, forward primer |
| Human-IL-1β-rev | TTTTTGCTGTGAGTCCCGGAG | Quantitative PCR for human IL-1β, reverse primer |
| Human-TNF-α-for | GAGGCCAAGCCCTGGTATG | Quantitative PCR for human TNF-α, forward primer |
| Human-TNF-α-rev | CGGGCCGATTGATCTCAGC | Quantitative PCR for human TNF-α, reverse primer |
| Human-CXCL2-for | ATTCTCAACACTCCAAACTGTGC | Quantitative PCR for human CXCL2, forward primer |
| Human-CXCL2-rev | ACTTTAGCTTCGGGTCAATGC | Quantitative PCR for human CXCL2, reverse primer |
| Human-IL-6-for | AGGAAGGGCCGTCTATCAATC | Quantitative PCR for human IL-6, forward primer |
| Human-IL-6-rev | CACTGTCACTTCGTGGAACTG | Quantitative PCR for human IL-6, reverse primer |

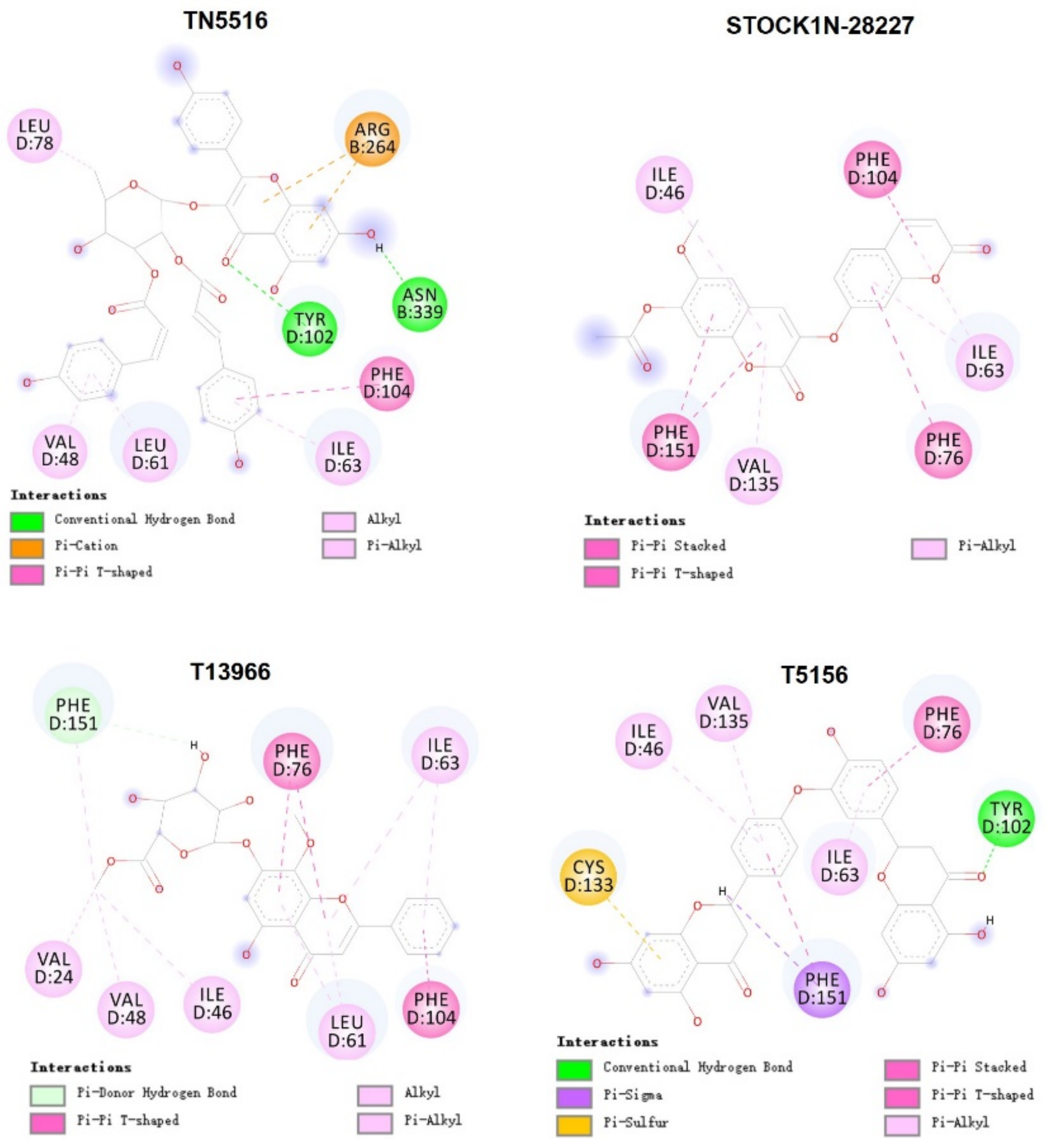


**Fig. (S1).** Molecular docking of selected compounds with TLR4/MD2 complex. Candidate compounds (TN5516, STOCK1N-28227, T13966, and T5156) target TLR4/MD2 active sites, the main forces include conventional hydrogen bonds (green), Pi-sulfur bonds (yellow), Pi-stacking (pink) and Pi-alkyl (purple).

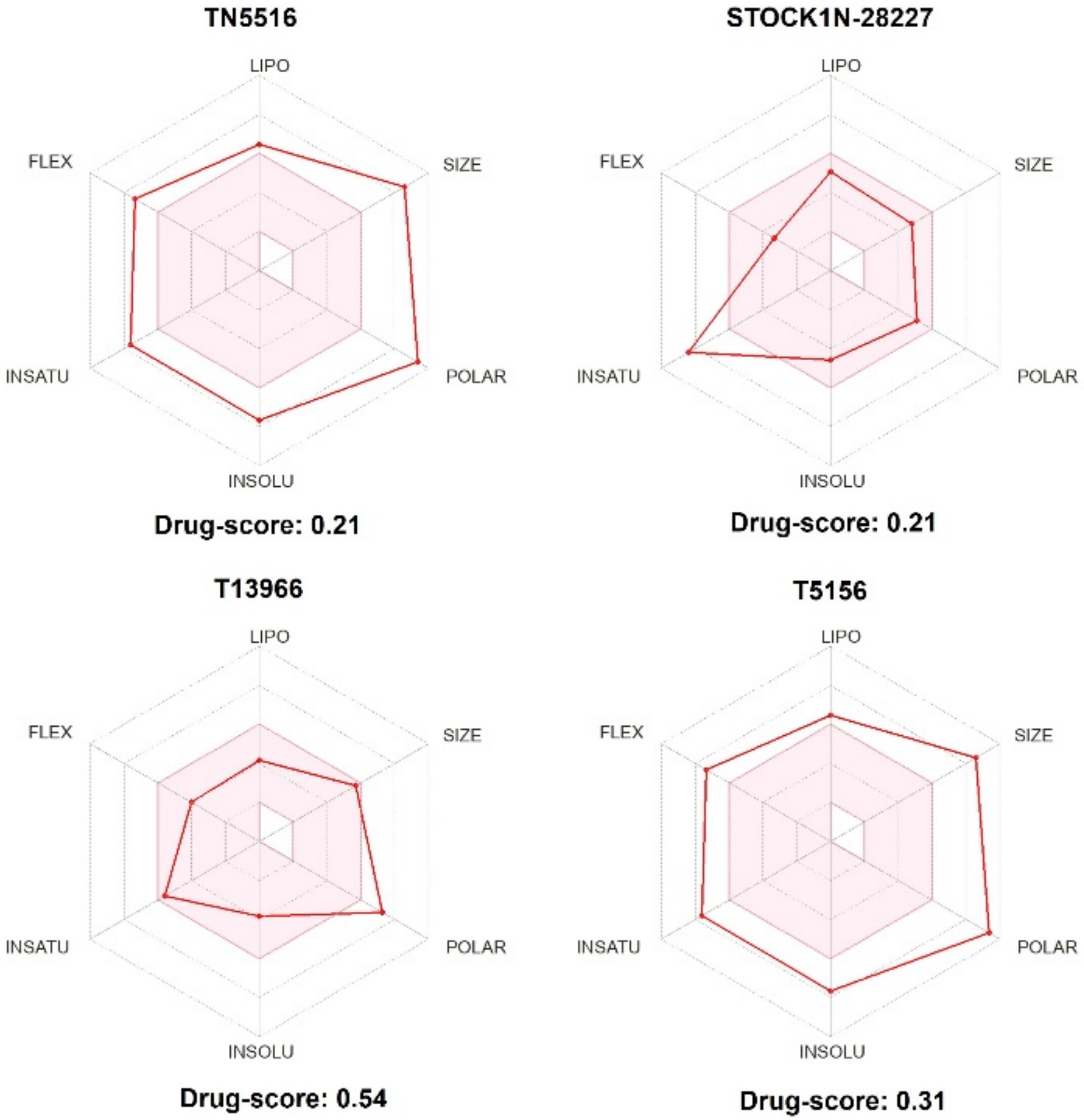


**Fig. (S2).** Drug scores of top7-10 candidate compounds and radar chart of their physicochemical properties. SIZE (size), POLAR (polarity), INSATU (unsaturation), INSOLU (insolubility), FLEX (flexibility), and LIPO (lipid solubility). The drug score of each compound is given below the radar chart.
